# Is endemic political corruption hampering provision of ART and PMTCT in developing countries?

**DOI:** 10.7448/IAS.17.1.18568

**Published:** 2014-05-02

**Authors:** Wing Young Nicola Man, Heather Worth, Angela Kelly, David P Wilson, Peter Siba

**Affiliations:** 1 International HIV Research Group, School of Public Health and Community Medicine, University of New South Wales, Sydney, Australia; 2 Faculty of Health Sciences, University of Sydney, Sydney, Australia; 3 Papua New Guinea Institute of Medical Research, Goroka, Papua New Guinea; 4 Kirby Institute, University of New South Wales, Sydney, Australia

**Keywords:** antiretroviral therapy, prevention of mother-to-child transmission, leadership, governance, corruption

## Abstract

**Introduction:**

Leadership is a key factor in the success of HIV prevention and treatment. Positive HIV-related outcomes are also affected by funding levels for HIV, health sector resources, disease burden and the socio-economic environment. Leadership on HIV as well as these other factors are affected by the quality of political governance of the country, which may be an overarching factor that influences the making of effective responses to the HIV epidemic.

**Aim:**

The aim of the study was to investigate the association between quality of political governance, on one hand, and coverage of antiretroviral therapy (ART) and prevention of mother-to-child transmission (PMTCT), on the other, in low- to middle-income countries.

**Methods:**

This investigation was carried out through a global review, online data sourcing and statistical analyses. We collected data on health burden and resources, the socio-economic environment, HIV prevalence, ART and PMTCT coverage and indicators of political governance. Outcome variables were coverage of ART (from 2004) and PMTCT (from 2007) to 2009 as a percentage of persons needing it. Potential predictors of treatment coverage were fitted with a baseline multilevel model for univariable and multivariable analyses.

**Results:**

Countries with higher levels of political voice and accountability, more political stability and better control of corruption have higher levels of ART coverage but not PMTCT coverage. Control of corruption (in standard deviation units) had a strong association with ART (AOR=1.82, *p*=0.002) and PMTCT (AOR=1.97, *p*=0.01) coverage. Indicators of economic development were not significant when control of corruption was included in the multivariable regression model. Many countries in all income groups had high ART but not PMTCT coverage (e.g. Mexico, Brazil and Romania in the upper-middle-income group; Papua New Guinea and Philippines in the lower-middle-income group; and Cambodia, Laos and Comoros in the low-income group). Very few low-income countries (notably, Haiti and Kenya) had high PMTCT coverage.

**Conclusions:**

Our research found a significant relationship between quality of political governance and treatment coverage. Measures and policies for improving the quality of political governance should be considered as a part of HIV programme implementation to more effectively improve the welfare of people living with HIV, particularly mothers living with HIV and their babies.

## Introduction

From the beginning of the HIV epidemic, it has been recognized that leadership is a key factor in the success of HIV prevention and treatment at the community, national and global levels [1–3]. As Piot and Seck [4] state, a central component in changing the epidemic's course is the willingness of leaders, at both national and provincial levels, to make a political commitment to respond to the epidemic, to acknowledge the AIDS crisis and to swiftly implement interventions even in the face of opposition. This political commitment is the extent to which leaders support HIV as a priority on the national, provincial or local agenda as evidenced through actions that include legislative, financial and programmatic components. It is also how leaders interact with and support those most affected by HIV, including the private sector, civil society and local nongovernment organizations (NGOs). As Gruskin and Tarantola [5] argue, concrete steps are needed to enhance political commitment at all levels in order to escalate the responses to HIV on the ground and within the environments in which they unfold. While enhancing leadership on mounting effective responses to HIV has come to be recognized as a critically important endeavour as the HIV pandemic moves into its fourth decade, it is also important to recognize that the willingness and effectiveness of a government to make such responses may be influenced by the quality of political governance in the country [1]. Others have shown that there is a relationship between HIV-related health coverage and funding levels, health sector resources, disease burden and socio-economic development [6–9]. Both political leadership on HIV and other factors affecting HIV-related health coverage are affected by the quality of political governance of the country [1,10], possibly making it an overarching factor that influences effective responses to HIV.

Political commitment is likely to be most meaningful where concrete policy and programmatic interventions are championed. A major component of both the global and governmental responses to HIV during the time period of this article is the provision of antiretroviral therapy (ART) to reduce AIDS-related deaths, to ensure the continued health of those diagnosed with HIV and to reduce mother-to-child transmission of HIV. UN Millennium Development Goal 6b (to achieve, by 2010, universal access to treatment for HIV/AIDS for all those who need it) and the “3 by 5” initiative, launched in 2003 by the World Health Organization (WHO) and the Joint United Nations Programme on HIV and AIDS (UNAIDS), were billed as a step towards ensuring that HIV/AIDS treatment was universally accessible to all who needed it [11]. In the 2005 World Summit, countries committed to “a massive scaling up of HIV prevention, treatment and care with the aim of coming as close as possible to the goal of universal access to treatment by 2010 for all who need it” [12]. While governments’ commitments have been variable, there is evidence that in a number of countries there has been a growing commitment on the part of governments to take a leading role in responding to the epidemic [13–16].

The main aim of this study was to investigate the quality of political governance as a determinant of coverage of ART and prevention of mother-to-child transmission (PMTCT) among those in need. ART and PMTCT rollouts have been significantly quantified, and we are arguing that the quality of political governance is an overarching factor that influences treatment coverage.

The measures of political governance used in this study are derived based on scores from a range of sources in the international community, and they apply across broad areas of the society (i.e. not just to health and HIV). Using these measures, we hypothesize that good political governance improves access to HIV treatment and prevention as measured by ART and PMTCT coverage. This has been done by a global review, online sourcing of country-level data and statistical analyses.

## Materials and methods

### Data collection

Countries with low to upper middle income as defined by the World Bank [17] were chosen for collection of country-level data on HIV, health burden and resources, socio-demography, economy, development and political governance from various sources towards the end of 2011.

#### Outcomes: ART and PMTCT coverage

The outcome variables chosen for analyses were:

1. ART coverage from 2004 to 2009, as a percentage of the number of persons living with HIV and needing ART, as estimated by UNAIDS: The need for ART is defined by the criteria of having clinical signs of severe immune suppression and/or a CD4 cell count <200 cells per microliter [18]. This is United Nations General Assembly Special Session (UNGASS) indicator 4 [13].
2. PMTCT coverage from 2004 to 2009, as a percentage of the estimated number of pregnant women living with HIV: This is UNGASS indicator 5, for which those who had PMTCT are defined as pregnant women living with HIV who received antiretroviral medicines in the last 12 months [19].

Estimates of coverage for ART and PMTCT were collected from UNAIDS data sets as given in the biennial Global Progress Reports, the latest of which was published in 2010 reporting data for the year 2009 [20]. Data from AIDSinfo on ART coverage as a percentage of those with a CD4 cell count <350 cells per microliter are used to impute missing values on ART coverage from the progress reports (Table 1; and see the “Data transformation and missing data imputation” section) [21].

**Table 1:** Data source and linear regression models for imputation of time-varying variables

	Primary data source	Secondary data source	Single imputation model (deterministic)	Multiple imputation model (stochastic)
**Outcome variables**				
ART coverage (%) of those with CD4 cell count <200 cells/µl	UNAIDS (of those with CD4 <200) [20]	WHO (of those with CD4 <350) [21]	ART coverage (%) of those with CD4 <350	Not imputed
PMTCT coverage (%)	UNAIDS [20]	–	Not imputed	Not imputed
**Health variables**				
HIV prevalence	UNAIDS [20]	–	Country, year and country by year interaction (interpolated)	Not imputed
Log(international non-HIV health funding USC|/capita)	QWID [35] b	–	Not imputed	Not imputed
Log(international HIV funding, USC|/capita)	QWID [35] b	–	Not imputed	Not imputed
Received PEPFAR funding	PEPFAR [25,26]	–	Not imputed	Not imputed
Received Global Fund for HIV	Global Fund [27]	–	Not imputed	Not imputed
Logit(Skilled birth attendants at delivery, %)	WDI [31] a,c	–	Country, year and country by year interaction (interpolated)	Main predictors,^e^ health resource indicators, logit(HDI), logit(GII) and logit(adult literacy)
Logit(Pregnant women receiving antenatal care, %)	WDI [31] a,c	–	Country, year and country by year interaction (interpolated)	Main predictors,^e^ health resource indicators, logit(HDI), logit(GII) and logit(adult literacy)
**Socio-economic variables**				
Log(GDP/capita/year [by purchasing power parities])	Gapminder [32]	WDI [31] a	Not imputed	Country, year, outcome variables and log(GDP/per capita) variables from WDI
Logit(Adult literacy [% of those ≥15 years])	UNESCO [31] a	UNESCO [32] d	Country, year and country by year interaction (interpolated)	Main predictors,^e^ WGI^f^ and socio-economic development indicators
Logit(Access to sanitation [% of population])	UNDP [31] a	–	Country, year and country by year interaction (interpolated)	Main predictors,^e^ WGI^f^ and socio-economic development indicators
Logit(Gender inequality index)^a^	UNDP [33]	–	Country, year and country by year interaction (interpolated)	Main predictors,^e^ WGI^f^ and socio-economic development indicators
**Political variables**				
Logit(Democracy score)	Freedom House [23]	–	Not imputed	Main predictors^e^ and WGI^f^
Political voice and accountability	Kaufmann *et al*. [22]	–	Not imputed	Not imputed
Political stability	Kaufmann *et al*. [22]	–	Not imputed	Not imputed
Political control of corruption	Kaufmann *et al*. [22]	–	Not imputed	Main predictors^e^ and WGI^f^
Rule of law	Kaufmann *et al*. [22]	–	Not imputed	Not imputed
Government effectiveness	Kaufmann *et al*. [22]	–	Not imputed	Not imputed
Regulatory quality of government	Kaufmann *et al*. [22]	–	Not imputed	Main predictors^e^ and WGI^f^

a WDI = World Development Indicators from World Bank.

b Query Wizard for International Development and Statistics (QWIDS) obtained through Institute for Health Metrics and Evaluation.

c Compiled from household surveys, including Demographic and Health Surveys by Macro International and Multiple Indicator Cluster Surveys by the United Nations Children's Fund (UNICEF), and UNICEF's The State of the World's Children 2010.

d From Gap Minder.

e The main predictors for multiple imputation are country, year, log(GDP/capita [PPP]) and HIV-related outcome variable. As this is created as a generic data set for the analysis of HIV-related outcomes, these outcomes include treatment coverage (ART and PMTCT), condom use and HIV knowledge in the general and vulnerable populations (e.g. sex workers and men who have sex with men).

f WGI are the six political governance indicators from Kaufmann *et al*. [22]. These are: voice and accountability; political stability and absence of violence; government effectiveness; regulatory quality; rule of law; control of corruption.

#### Exposure of interest: political governance

Six aggregate indicators of the quality of political governance derived by Kaufmann *et al*. were used [22]:

1. political voice and accountability – indicates the ability of a country's citizens to participate in selecting their government;
2. political stability (and lack of politically motivated violence) – indicates the likelihood that the government will be destabilized or overthrown by unconstitutional or violent means;
3. control of corruption – indicates the extent to which public power is exercised for private gain, as well as “capture” of the state by elites and private interests;
4. rule of law – indicates the confidence and abidance of agents in the laws or rules of the society, in particular the quality of contract enforcement, property rights, the police, and the courts, as well as the likelihood of crime and violence;
5. government effectiveness – indicates the quality of public and civil services and their degree of independence from political pressures, the quality of policy formulation and implementation, and the credibility of the government's commitment to such policies; and
6. regulatory quality of government – indicates the ability of the government to implement sound policies and regulations that facilitate private sector development.

Each indicator is based on the views of survey respondents and of public, private and NGO sector experts worldwide. They were taken from several hundred individual underlying variables in a wide variety of existing data sources, and combined into a single score for each of these six indicators using the Unobserved Components Model, which allows the standardization of data from diverse sources into comparable units [22]. This set of indicators has been widely used among policy makers and academics because of its very broad country coverage over a range of years and its ability to smooth out some of the idiosyncrasies of individual measures of governance through its use of many different data sources [23]. Since there are no consensus definitions on governance indicators, its construct validity is difficult, if not impossible, to evaluate. However, it demonstrates convergent validity as each of the six aggregate indicators shows high correlation with the individual sources it derives from. The scores were standardized to one standard deviation unit within each year. However, the reported standard deviation in this study is smaller as high-income countries are excluded (see later in Table 2). Democracy scores from Freedom House with imputation of missing scores from the Polity IV Project were obtained from Gothenburg University [23].

**Table 2:** Characteristics of countries in analysis^a^

		HIV prevalence					
				
		<0.5%	≥0.5 & <1.5%	≥1.5 & <5%	≥5%	Overall	
			
	*n* b	Mean±standard deviation					*P* e
***Time-varying variables*** **b**		***n*** **=295**	***n*** **=202**	***n*** **=109**	***n*** **=90**	***n*** **=696**	
**Outcome variables**							
ART coverage (%)	678	36.6±31.5	38.4±26.6	23.9±17.0	34.1±24.7	34.7±27.8	**0.03** **f**
PMTCT coverage (%)	348	35.5±35.9	46.4±36.5	19.3±21.7	36.2±26.2	34.6±32.2	0.27^f^
**Health variables**							
International non-HIV health funding USC|/capita	696	96.6±564	55.6±109	33.9±38.3	25.2±17.8	65.7±373.2	0.90^f^,^g^
Had international HIV health funding^c^	696	87.8% (259)	83.2% (168)	100% (109)	100% (90)	91.5% (626)	–
International HIV funding, USC|/capita	696	6.9±25.9	21.1±66.6	18.7±18.7	79.0±153	22.2±71.6	**<0.001** **f**,**g**
International HIV funding, USC|/person living with HIV	631	118±529	33.0±96.9	13.9±14.0	8.2±10.3	62.7±354	**0.04** **f**,**g**
Received PEPFAR funding^c^	696	4.1% (12)	9.9% (20)	29.4% (109)	63.3% (57)	17.4% (121)	–
Received Global Fund for HIV^c^	696	72.5% (214)	64.4% (130)	89.9% (98)	84.4% (76)	74.4% (518)	–
Skilled birth attendants at delivery (%)	199	85.4±23.2	81.3±23.0	57.7±26.4	60.6±13.5	77.8±25.2	**<0.001** **f**
Pregnant women receiving antenatal care (%)	161	86.4±15.9	88.8±15.3	84.0±17.0	88.0±7.4	86.8±15.2	0.25^f^
**Socio-economic variables**							
GDP/capita/year	693	5900±3743	5590±4583	2188±2176	3647±4160	4933±4109	0.19^f^,^g^
Adult literacy (% of those ≥15 years)	289	86.3±14.8	76.6±22.9	56.8±18.9	76.4±12.4	78.1±20.6	**0.01** **f**
Access to sanitation (% of population)	218	71.1±24.7	62.5±30.0	37.4±26.9	41.7±15.4	58.6±29.0	**0.005** **f**
Access to improved water source (% of population)	217	87.3±13.3	79.2±19.8	70.6±16.9	72.8±14.8	80.0±17.4	0.07^f^
Gender inequality index^d^	175	4.31±1.14	5.05±1.43	5.74±0.85	5.85±0.61	4.97±1.28	**<0.001** **f**
**Political variables**							
Democracy score	692	5.75±2.91	6.62±2.73	5.22±2.23	5.52±2.54	5.89±2.76	**0.03** **f**
Political voice and accountability	696	−0.42±0.77	−0.25±0.88	−0.71±0.66	−0.38±0.66	−0.41±0.79	0.34^f^
Political stability	696	−0.42±0.83	−0.38±0.97	−0.82±0.77	−0.24±0.78	−0.45±0.87	**0.04** **f**
Political control of corruption	695	−0.43±0.54	−0.43±0.73	−0.82±0.42	−0.51±0.61	−0.50±0.61	0.07^f^
Rule of law	696	−0.42±0.57	−0.51±0.78	−0.94±0.48	−0.56±0.61	−0.55±0.65	0.19^f^
Government effectiveness	696	−0.36±0.55	−0.40±0.74	−0.92±0.52	−0.49±0.61	−0.48±0.64	0.17^f^
Regulatory quality of government	695	−0.35±0.68	−0.39±0.81	−0.79±0.48	−0.46±0.65	−0.44±0.71	0.19^f^
***Time-constant variables*** **b**		***n*** **=58**	***n*** **=39**	***n*** **=19**	***n*** **=14**	***n*** **=130**	
**Health variables**							
HIV DALY/100,000 in 2004	130	1.02±1.50	8.41±7.72	32.8±19.9	158±93.5	24.8±56.8	**<0.001** **g**,**h**
TB DALY/100,000 in 2004	130	4.71±12.6	5.77±5.43	14.1±7.34	13.1±5.16	7.31±10.2	**<0.001** **g**,**h**
Maternal and child DALY/100,000 in 2004	129	4.10±3.74	8.74±10.33	21.7±11.9	11.8±5.60	8.93±9.82	**<0.001** **g**,**h**
Non-HIV DALY/100,000 in 2004	130	247±391	272±148	459±178	302±88.9	291±291	**<0.001** **g**,**h**
**Geographical and ethno-linguistic diversity variables**							
Ethno-linguistic fractionalization^d^	130	3.93±2.66	4.01±3.24	6.34±3.47	4.29±2.82	4.35±3.07	**0.02** **h**
Regions^b^	130						–
Asia-Pacific		34.5% (20)	12.8% (5)	0% (0)	0% (0)	21.6%(29)	
Europe and Central Asia		29.3% (17)	7.7% (3)	0% (0)	0% (0)	14.2%(19)	
Latin America and the Caribbean		19.0% (11)	41.0% (16)	15.8% (3)	0% (0)	21.6%(29)	
Middle-East and North Africa		13.8% (8)	0.0% (0)	5.3% (1)	0% (0)	8.2%(11)	
Sub-Saharan Africa		3.4% (2)	38.5%(15)	78.9% (15)	100% (14)	34.3%(46)	

a Only countries with estimate(s) of HIV prevalence and one or more of the outcome variables estimated (ART coverage or PMTCT coverage) are included in this study.

b This is the total number of observations for each country and year combination for the longitudinal variables and the total number of countries for the cross-sectional variables.

c Column percentage (and *n*) is given instead of the mean±standard deviation.

d Rescaled so that the plausible range is from 0 to 10 (original plausible range was from 0 to 1).

e Values in bold indicate statistical significance at p<0.05.

f Tested using a multilevel model fitting HIV prevalence categories and year, and clustered by country.

g Tested on the log_10_-transformed variable.

h Tested using a linear regression model fitting HIV prevalence categories.

#### Control covariates

Estimates of HIV prevalence were collected from UNAIDS data sets as given in Global Progress Reports and used to account for differences in the resources necessary to achieve high treatment coverage and for differences in attention that HIV/AIDS might receive in public policy debates [20]. Countries were grouped into four categories based on HIV prevalence levels for descriptive analyses: very low prevalence at <0.5%, low prevalence at ≥0.5 and <1.5% prevalence, high prevalence at ≥1.5 and <5% and very high prevalence at ≥5%. Where estimates of HIV prevalence were not available, UNGASS country progress reports were used for determining the HIV prevalence group in which a country belonged [24]. HIV prevalence percentage was fitted as linear and quadratic covariates, as preliminary analyses indicated that treatment coverage decreased as HIV prevalence increased somewhat; however, coverage increased as HIV prevalence increased further. This would be expected if resource depletion due to high demand was the factor impeding treatment coverage, but coverage could increase at high HIV prevalence levels when there was increasing political will, as well as funding to provide treatment to the increasing numbers of citizens affected by HIV.

Other potential control covariates are:

1. Non-HIV disability-adjusted life years (DALYs) at baseline in 2004: These variables give an indication of health burden in the country that can compete with HIV health care provision in the allocation of health care resources.
2. International non-HIV funding per capita, international HIV funding per person living with HIV, being a President's Emergency Plan for AIDS Relief (PEPFAR) focus country [25,26], receiving Global Fund for HIV/AIDS support [27], the percentage of pregnant women attending at least one antenatal visit and the percentage of skilled birth attendants at delivery from WHO data [28]: These are included as indicators of health resource availability in terms of funding, infrastructure and workers for health care which can facilitate ART and PMTCT coverage in the country. The availability of resources for the provision of non-HIV health services (e.g. international non-HIV funding) also means that there is less competition for domestic health resources which can be diverted towards HIV health care provision.
3. GDP per capita (by purchasing power parities), adult literacy rate (percentage among ≥15 year olds from United Nations Educational, Scientific and Cultural Organization (UNESCO)) and gender inequality index: These are indicators of socio-economic development which indicate the availability of domestic resources such as income and workforce skills that are needed for ART and PMTCT provision. The gender inequality index indicates the status of women in the society, which would particularly affect PMTCT coverage.
4. Geographical region and ethno-linguistic fractionalization: Certain geographical regions are more responsive to the scale-up in provision of ART and PMTCT coverage, which could be due to a more conducive regional political climate for such political actions. Ethno-linguistic fractionalization has been shown to affect provision of public services, including health care; hence, it may affect ART and PMTCT coverage [29,30].

Longitudinal databases obtained from the World Bank and the Gapminder Foundation were used as the major sources of data for this study [31,32]. These two comprehensive databases included indicators collated from various organizations, including WHO, UNESCO and the United Nations Development Programme (UNDP). Table 1 shows the sources of data used in this study.

Time-constant measures of ethno-linguistic fractionalization were obtained from Desmet *et al*. [29], and ELF9 in their data set was used in the analyses. The data on gender inequality index presented in the Human Development Report 2011 were obtained from UNDP [33]. Data on international health funding and on estimated population-level accumulated DALYs (as time-constant variables estimated in 2004 only) were obtained from the Institute for Health Metrics and Evaluation [34]. International health funding data in the IHME database were obtained from the Query Wizard for International Development and Statistics (QWIDS) of the Organisation for Economic Co-operation and Development (OECD) [35]. Data on DALYs were estimated by WHO in 2004 based on estimated incidence and/or prevalence of diseases or health conditions and the estimated proportion of the population who are treated out of those with the disease or condition. From these data, the estimated impact of the disease in terms of years of life lost (YLLs) due to premature mortality and years lost due to disability (YLDs) were calculated and summed to obtain DALY estimates [36].

### Statistical analysis

Stata version 12.1 (Stata Corp., College Station, TX) was used for data management and all statistical analyses.

#### Data transformation and missing data imputation

There were some missing observations in many potential predictors that drastically reduced sample sizes and may potentially lead to biases in the estimates. For this reason, missing observations in the predictor variables were imputed for subsequent statistical analyses. Transformations as appropriate to the variable were carried out before missing data imputation, as the procedure requires variables with a normal distribution and unbounded ranges. Variables with bounded ranges (e.g. percentages and scores) were logit-transformed before imputation. The logit-transformed predictor variables were back-transformed and rescaled to range from 0 to 10 for statistical analyses, so that regression coefficients can be interpreted as per a 10% increase in the scale range. Variables with skewness greater than 1 were log_10_-transformed (e.g. international health funding, GDP per capita and HIV prevalence) for imputation and statistical analyses, except for HIV prevalence which was back-transformed after imputation for statistical analysis on the original percentage scale.

Where more than one data source existed for an indicator, the source containing the most complete records was used as the main variable for the indicator. The remaining missing values of the variables with more than one data source were filled in from other data sources if the difference in the means was <2% of the standard deviation of the main variable and there was a strong correlation between the indicator from the two sources (*r*>0.95). Otherwise, these variables from other data sources were entered into missing data imputation models. Multiple-chained (stochastic) imputation with 50 replicates was performed in Stata on all predictor variables with missing observations using linear regression models. Several models of chained imputation were tested, and comparison of results in the univariable analyses indicated that the estimates were generally similar. For the bounded predictor variables with more than 50% of their observations missing, missing values for countries with at least two data points were firstly imputed deterministically with a single imputation linear regression model within the range of years in which observed data were available (i.e. interpolated). This deterministic imputation of data was performed because of reduced statistical efficiency (or increased error in the estimates) if these were only imputed stochastically. The imputation models used are shown in Table 1.

For percentage of ART coverage, data from both UNAIDS and WHO were obtained. Data from UNAIDS generally estimated a higher level of ART coverage compared with data from WHO, particularly in the later years. Data from UNAIDS were used as the main variable, and where available, missing data in the UNAIDS variable were deterministically imputed from WHO data in the World Health Statistics Report as presented in AIDSinfo [21,28]. The outcomes, percentage of ART and PMTCT coverage, were fitted as logit-transformed variables, and the back-transformed regression coefficients can be interpreted as the odds ratio (OR) [37]. It yields regression coefficients conditional on the means, which is generally better than untransformed proportions [38]. Appropriate back transformation can also yield predicted proportions with confidence intervals bounded within the plausible range (i.e. between 0 and 1) [38]. While there are better regression models for proportions as outcome data (e.g. beta regression), such models are not readily available for multilevel modelling in standard statistical packages, and the regression coefficients may be difficult to interpret.

#### Statistical model and potential predictors

A baseline multilevel model was used for analysing the determinants of ART and PMTCT coverage. This baseline model fitted year of estimation and HIV prevalence (linear and quadratic covariates) as fixed effects and random intercept for country and random slope for year. Multilevel models are used as they take into account similarities in measures within a cluster of observations by modelling the within-cluster and between-cluster variations with the fitting of the random intercept for each cluster (i.e. country in this study). Multilevel models are also suitable for the analyses of longitudinal panel data [39]. In longitudinal data analyses, the random slope for year (as a measure of time) allows for variation in the increase (or decrease) in ART and PMTCT coverage between countries over time, as well as allows for significant differences between countries in change over time to be tested.

Variables with *p*<0.10 in the univariable analyses using the baseline multilevel model were entered and tested in a multivariable multilevel model. Variables with *p*<0.10 in the multivariable model were retained in the final model. As GDP per capita (log-transformed) is an important determinant of the resources a country has for providing ART and PMTCT, it is kept in the final model regardless of its statistical significance.

## Results

Characteristics of low- and middle-income countries, by HIV prevalence category, are shown in Table 2. One hundred and four countries out of the 130 countries in this study had estimates of ART coverage for every year from 2004 to 2009 in this study. Estimates of PMTCT coverage were less complete in earlier years, with fewer than 50 countries having estimates of PMTCT within each year before 2008; this increased to 74 countries in 2008 and 108 countries in 2009. Countries in the high-HIV-prevalence category (≥1.5 and <5%) had lower ART and PMTCT coverage in comparison with countries in other HIV prevalence categories. This could be because the countries in the category with HIV prevalence ≥1.5 and <5% had very limited resources and low levels of development, as indicated by their low GDP and low levels of human development as well as worse ratings on political governance, even in comparison to countries in the very-high-prevalence category.

### Analysis on potential determinants of ART and PMTCT coverage

Results from univariable analyses for associations between ART and PMTCT coverage and each potential predictor variable are shown in Table 3. Overall ART coverage improved significantly from 16.7% in 2004 to 56.2% in 2009, and PMTCT coverage improved significantly from 17.1% in 2004 to 45.2% in 2009. Linear association with HIV prevalence is interpreted as the OR of a 1% increase in treatment coverage with a 1% increase in HIV prevalence when HIV prevalence is at 0%, where the predicted odds of decrease in coverage is steepest (i.e. the smallest OR). It was not significant for both ART and PMTCT coverage in the baseline multilevel model. However, there is a significant (quadratic) rate of increase in ART coverage as HIV prevalence increased. Higher levels of socioeconomic development are associated with better treatment coverage, but these, including log(GDP/capita), were not statistically significant in the multivariable analyses. The amount of international funding for HIV is not a significant predictor of treatment coverage; however, being a PEPFAR focus country is associated with better ART coverage in both the univariable and multivariable analyses.

**Table 3:** Univariable analysis for association with percentage of ART and PMTCT coverage (logit-transformed; based on UNAIDS estimates)

	ART coverage (*n*=678)		PMTCT coverage (*n*=348)	
		
	Adjusted OR (95% CI)	*P* (Wald χ^2^)^e^	Adjusted OR (95% CI)	*P* (Wald χ^2^)^e^
**Base model**				
Year	**1.65 (1.55, 1.76)**	**<0.001 (248)**	**1.49 (1.37, 1.61)**	**<0.001 (90.4)**
HIV prevalence, %				
Linear	0.86 (0.73, 1.00)^b^	0.05 (3.7)^b^	0.88 (0.70, 1.12)^b^	0.31 (1.1)^b^
Quadratic	**1.01 (1.00, 1.01)** ^**b**^	**0.04 (4.1)** ^**b**^	1.01 (1.00, 1.02)^b^	0.17 (1.9)^b^
Random effects				
Intercept (or country)	–	**<0.001 (524)** **c**	–	**<0.001 (268)** **c**
Year	–	**<0.001 (159)** **d**	–	**0.02 (8.21)** **d**
**Health variables**				
log(TB DALY/10,000 in 2004)	**0.35 (0.24, 0.50)**	**<0.001 (31.4)**	**0.26 (0.14, 0.48)**	**<0.001 (18.5)**
log(maternal and child DALY/10,000 in 2004)	**0.38 (0.24, 0.62)** ^**b**^	**<0.001 (15.4)** ^**b**^	**0.13 (0.06, 0.28)**	**<0.001 (27.4)**
log(non-HIV DALY/10,000 in 2004)	**0.09 (0.03, 0.27)**	**<0.001 (18.5)**	**0.02 (<0.01, 0.14)**	**<0.001 (16.6)**
log(international non-HIV health funding, USC|/capita)	**1.68 (1.35, 2.10)**	**<0.001 (20.9)**	1.06 (0.67, 1.69)	0.80 (0.07)
log(international HIV funding, USC|/ HIV-infected person)	0.97 (0.85, 1.12)^b^	0.71 (0.13)^b^	1.00 (0.77, 1.30)^b^	0.99 (<0.01)^b^
Received Global Fund for HIV	1.02 (0.84, 1.23)	0.87 (0.03)	1.00 (0.68, 1.46)	0.99 (<0.01)
Received PEPFAR focus country funding	**1.52 (1.06, 2.18)**	**0.02 (5.1)**	0.86 (0.47, 1.58)	0.63 (0.24)
Skilled birth attendants at delivery (per 10%)	**1.11 (1.01, 1.21)**	**0.03 (4.9)**	**1.30 (1.12, 1.52)**	**0.001 (11.2)**
Pregnant women receiving antenatal care (per 10%)	**1.15 (1.02, 1.30)**	**0.02 (5.2)**	1.27 (0.97, 1.65)	0.08 (3.06)
**Socio-economic and geographical variables**				
Log(GDP/capita)	**3.38 (1.92, 5.95)** b	**<0.001 (17.9)** b	**11.6 (4.78, 28.2)** ^**b**^	**<0.001 (29.4)** ^**b**^
Adult literacy rate (per 10%)	**1.19 (1.06, 1.34)** ^**b**^	**0.003 (8.5)** ^**b**^	**1.40 (1.15, 1.72)** ^**b**^	**<0.001 (10.6)** ^**b**^
Access to sanitation (per 10%)	**1.11 (1.02, 1.21)** ^**b**^	**0.02 (5.4)** ^**b**^	**1.29 (1.12, 1.49)** ^**b**^	**<0.001 (12.8)** ^**b**^
Gender inequality index^a^	0.80 (0.62, 1.03)^b^	0.08 (3.2)^b^	**0.61 (0.41, 0.90)** b	**0.01 (6.3)** b
Ethno-linguistic fractionalization^a^	**0.85 (0.78, 0.92)**	**<0.001 (17.5)**	**0.87 (0.76, 1.00)**	**0.04 (4.1)**
Regions (reference: Asia-Pacific)		**<0.001 (28.2)**		**<0.001 (33.6)**
Europe and Central Asia	1.05 (0.50, 2.22)	0.89 (0.02)	3.45 (0.97, 12.2)	0.06 (3.7)
Latin America and the Caribbean	**3.71 (1.90, 7.24)**	**<0.001 (14.8)**	**9.53 (3.04, 29.9)**	**<0.001 (14.9)**
Middle-East and North Africa	0.68 (0.26, 1.79)	0.44 (0.61)	0.18 (0.03, 0.99)	0.05 (3.9)
Sub-Saharan Africa	0.85 (0.41, 1.76)	0.65 (0.20)	0.97 (0.30, 3.18)	0.96 (<0.01)
**Political variables**				
Democracy score	**1.10 (1.04, 1.17)** ^**b**^	**0.001 (10.3)** ^**b**^	**1.23 (1.09, 1.38)**	**<0.001 (12.5)**
Political voice and accountability	**2.05 (1.62, 2.60)**	**<0.001 (35.1)**	**2.06 (1.35, 3.14)**	**<0.001 (11.1)**
Political stability	**1.96 (1.62, 2.36)**	**<0.001 (48.1)**	**1.72 (1.23, 2.41)**	**<0.001 (10.0)**
Political control of corruption	**2.64 (1.95, 3.55)** ^**b**^	**<0.001 (40.3)** ^**b**^	**3.14 (1.89, 5.22)**	**<0.001 (19.5)**
Rule of law	**1.81 (1.35, 2.45)**	**<0.001 (15.3)**	**2.68 (1.61, 4.46)**	**<0.001 (14.3)**
Government effectiveness	**1.83 (1.35, 2.48)**	**<0.001 (15.1)**	**3.33 (2.04, 5.43)**	**<0.001 (23.2)**
Regulatory quality of government	**1.37 (1.04, 1.82)** ^**b**^	**0.03 (4.9)** ^**b**^	**2.27 (1.42, 3.63)**	**0.001 (11.7)**

a Rescaled so that the plausible range is from 0 to 10 (original plausible range was from 0 to 1).

b Missing data are imputed for these variables; χ^2^ statistic is estimated from (*F* statistic)×(*df*_num_), where the numerator degrees of freedom, *df*_num_=1 for continuous covariates, is given instead.

c Likelihood ratio test of multilevel model with random intercept only against linear regression with fixed effects only.

d Likelihood ratio test of multilevel model with random intercept and slope of year against multilevel model with random intercept only.

e Values in bold indicate statistical significance at p<0.05.

There was a significant association of ART and PMTCT coverage with all indicators of political governance in Table 3. Countries with higher levels of voice and accountability to its people, more political stability, better control of corruption in the public sector, better rule of law in the society, better government effectiveness and better regulatory quality to facilitate private sector development had higher levels of ART and PMTCT coverage. Political control of corruption stayed statistically significant in the multivariable analyses for both ART and PMTCT coverage, with an adjusted OR of 1.82 and 1.97, respectively, for a one standard deviation unit change in political control of corruption (Table 4). Political stability, political voice and accountability, and regulatory quality of the government were also significantly associated with ART coverage. For regulatory quality, the association in the multivariable analysis is opposite in direction to that in the univariable analysis (i.e. a lower score on regulatory quality is associated with higher levels of ART coverage).

**Table 4:** Predictors of percentage of ART and PMTCT coverage in the final multilevel model (logit-transformed; based on UNAIDS estimates)^a^

	ART coverage (*n*=678)		PMTCT coverage (*n*=348)	
		
	Adjusted OR (95% CI)	*P* (Wald χ^2^)^c^	Adjusted OR (95% CI)	*P* (Wald χ^2^)^c^
**Year**	**1.61 (1.51, 1.71)**	**<0.001 (224)**	**1.43 (1.31, 1.55)**	**<0.001 (63.6)**
**Health variables**				
HIV prevalence, %				
Linear	0.97 (0.84, 1.13)	0.73 (0.12)	1.19 (0.95, 1.50)	0.12 (2.4)
Quadratic	1.00 (0.99, 1.01)	0.98 (<0.01)	0.99 (0.98, 1.00)	0.25 (1.3)
Log(international non-HIV health funding, USC|/capita)	**1.38 (1.11, 1.72)**	**0.004 (8.2)**	–	–
Received PEPFAR funding	**1.62 (1.15, 2.27)**	**0.006 (7.6)**	–	–
**Socio-economic and geographical variables**				
Log(GDP/capita)	1.57 (0.86, 2.87)	0.14 (2.2)	**6.80 (2.61, 17.7)**	**<0.001 (15.4)**
Linguistic fractionalization^b^	**0.92 (0.85, 0.98)**	**0.01 (6.1)**	–	–
**Political variables**				
Political control of corruption	**1.82 (1.24, 2.66)**	**0.002 (9.4)**	**1.97 (1.14, 3.41)**	**0.01 (5.9)**
Political stability	**1.42 (1.13, 1.78)**	**0.002 (9.3)**	–	–
Political voice and accountability	**1.40 (1.04, 1.88)**	**0.02 (5.0)**	–	–
Regulatory quality of government	**0.60 (0.43, 0.84)**	**0.003 (8.9)**	–	–

a Random intercept and random slope were fitted, but their statistical significance cannot be tested with multiple imputation.

b Rescaled so that the plausible range is from 0 to 10 (original plausible range was from 0 to 1).

c χ^2^ statistic is estimated from (*F* statistic)×(numerator degrees of freedom). P-values in bold indicate statistical significance at p<0.05.

**Figure 1:**
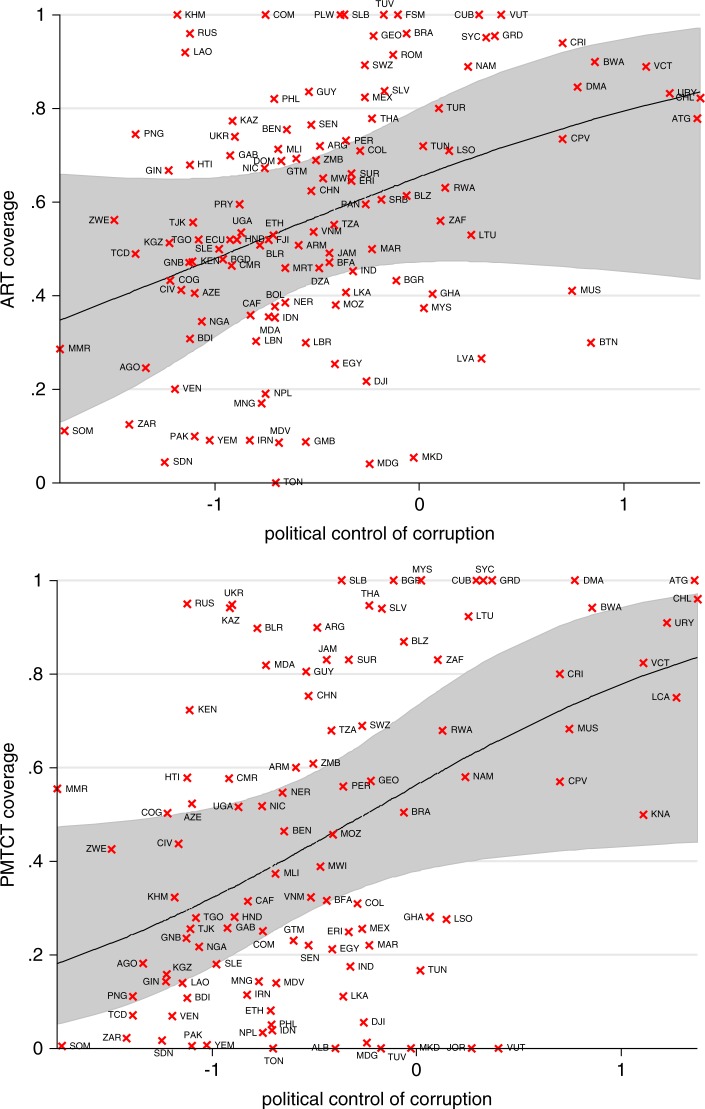
ART (top graph) and PMTCT (bottom graph) coverage against political control of corruption in 2009, and their fitted line and 95% confidence bands. Countries with significantly higher or lower treatment coverage (ART or PMTCT in the respective graphs) in the final models in Table 4 are indicated by filled symbols.

The relationships of ART (top figure) and PMTCT (bottom figure) coverage with control of corruption in 2009 in Figure 1 show that countries with higher income have lower levels of political corruption (those in the upper-middle-income category from World Bank data are indicated by black triangles, lower-middle income by blue circles and low income by red crosses). Countries with significantly higher or lower treatment coverage (ART or PMTCT in the respective graphs) in the final models in Table 4 are indicated by filled symbols. Almost all countries with both high control of corruption and high treatment coverage (ART and/or PMTCT) were upper-middle-income countries. However, a few countries in the upper-middle-income category had low treatment coverage, such as Venezuela (VEN) and Macedonia (MKD) with significantly lower than predicted ART and PMTCT coverage, and Lebanon (LBN) and Latvia (LVA) with significantly lower ART coverage (with no data on PMTCT coverage). Malaysia (MYS), Bulgaria (BGR) and Mauritius (MUS) had lower ART coverage than predicted, though BGR and MYS had 100% PMTCT coverage. Quite a few countries in all income groups had high ART coverage but not PMTCT coverage, such as Mexico (MEX), Brazil (BRA) and Romania (ROM) in the upper-middle-income group; Papua New Guinea (PNG) and Philippines (PHL) in the lower-middle-income group; and Cambodia (KHM), Laos (LAO) and Comoros (COM) in the low-income group. Only Haiti (HTI), Kenya (KEN), Tanzania (TZA) and Zambia (ZMB) as low-income countries had PMTCT coverage above the 99.7% confidence band.

## Discussion

This study utilizes data from the years after the launch of the “3 by 5” by WHO and UNAIDS in December 2003, to analyse HIV outcomes as measured by ART and PMTCT coverage as a percentage of estimated need for each country [11]. The outcome measures are logit-transformed as appropriate for variables such as proportions or percentages. The approach is in contrast to two other studies on the performance of countries which modelled untransformed or log-transformed outcomes in a cross-sectional analysis [1,40]. The advantage of using logit-transformed data is that predicted values do not exceed plausible ranges, which was an issue encountered in the Nattrass [14] study [38]. The multilevel models used in the analysis also enabled us to use data from all years while accounting for country-level clustering and to analyse trends in ART and PMTCT coverage over years.

A limitation of this study is the lack of consistently collected data on some of the indicators. Missing data imputation was carried out to enable analysis of the data for a wide range of variables over each year of analysis. However, most of the indicators with a large proportion of missing observations did not show a significant association with the outcomes possibly because they have higher type II error rates. Different countries may also use different criteria for defining an indicator. For example, the metadata showing the definition of adult literacy indicate that a whole range of criteria is used for defining literacy [41]. As a corollary, the measures of political governance are among one of the most complete indicator variables and are likely to be more robust than many of the other indicators as they are constructed from multiple sources in a rigorous manner; hence, this may explain in part their strong association with treatment coverage.

The association between good political governance and ART and PMTCT coverage may be mediated through other factors such as level of socio-economic development or income level that more directly influence treatment coverage. This does not mean, however, that we should just act on these other factors. Instead, it implies that efforts to improve the political governance of a country may have wide-ranging impacts on the welfare of people in low- and middle-income countries as discussed below. Conversely, a certain level of socio-economic development such as networks for open communication and income level can be conducive to improvement in the quality of political governance [42,43].

Control of corruption had a strong association with both outcomes. This should perhaps be an important issue to address, particularly when it comes to accountability and effective use of resources for HIV programmes, but perhaps also for other programmes for development. There has been increasing interest among health policy makers, planners and donors in how corruption affects not only the HIV response but also health care access and outcomes in general [44–47]. For example, the purposelessness and incompleteness of Russian health reforms affected the response to HIV and led to an increase in corruption, a decline in access to services and a loss of health workers in the public sector [48–50]. The indicators of economic development, including GDP per capita, were not significant when control of corruption was included in the multivariable regression model, and this could be because it is highly correlated with the level of economic development. Many scholars have discussed the nexus between large-scale resource development and HIV [51]. For example, while Nigeria and Papua New Guinea are rich in natural resources, both have high levels of corruption. Udoh *et al*. [52] argue that a lack of transparency and competency in government leadership affected the viability and effectiveness of Nigeria's HIV programme, and that corruption surrounding oil exploration contributed to HIV transmission. Certainly the data indicate that poverty per se is not the driver of HIV; the situation is more complicated. Relative newfound cash wealth in the hands of men and the poverty of women which lends itself to sexual risk taking is one driver of the epidemic in Papua New Guinea. These connections between corruption, economic development and HIV should perhaps be an important issue to address, particularly when it comes to accountability and the effective use of resources for HIV programmes, but perhaps also for other programmes for development.

Political voice and accountability were also significantly associated with ART coverage. Such an association can be anticipated in view of the level of activism and civil involvement in the push towards universal ART coverage, so that governments that respond to pressure from its people are more likely to increase their efforts to improve ART provision in their countries. Such activism or level of involvement of the civil society was not seen to such an extent for PMTCT coverage. A politically stable environment is also associated with ART and PMTCT coverage – governments may need a stable “platform” from which to deliver needed services to their people. There is also the possibility of a vicious cycle or feedback loop – more political instability means poorer health outcomes, which in turn lead to more instability. On the other hand, there are lessons from the Brazilian experience that indicate that when the national response is “owned” by the government, civil society, the media and, most importantly, people living with HIV, there is considerable success in rolling out ART and PMTCT programmes [44].

This country-level ownership of the epidemic means that governments such as those of Brazil and Thailand are prepared to issue compulsory licensing so as to secure affordable ART drugs [53–55]. In our model, the direction of association of the regulatory quality of the government with ART coverage was reversed when the regulatory quality was included in the multivariable model. Judicious use of such trade restrictions may well be an action that can ensure the procurement of the drugs at such prices as would make universal ART (or PMTCT) coverage reasonably achievable.

## Conclusions

Even though there are calls worldwide for better political governance in the response to HIV, there has been little empirical evidence of the efficacy of good government. Our research has found that there is a significant relationship between HIV treatment coverage and the quality of political governance, particularly citizens’ voice and the government's accountability to their people, political stability and control of public sector corruption. However, it is important to recognize that other factors such as socio-economic development and income levels that are interrelated with political governance also affect HIV-related outcomes. Ways in which a country and its leaders can be encouraged and/or facilitated to provide good political governance, such as measures and policies for controlling corruption, should be considered for integration into HIV programmes. Even though practical integration of such measures and policies to improve political governance may only impact particular sectors of the public service (e.g. health services), it may enable the programmes to go further towards improving the health and welfare of people living with HIV, particularly mothers living with HIV and their babies.
